# Euglycemic diabetic ketoacidosis following major vascular surgery is a new item on the differential for postoperative acidosis

**DOI:** 10.1016/j.jvscit.2021.10.006

**Published:** 2021-10-22

**Authors:** Clara M. Gomez-Sanchez, Bian X. Wu, Jeffrey E. Gotts, Robert W. Chang

**Affiliations:** a Division of Vascular and Endovascular Surgery, Department of Surgery, University of California San Francisco, San Francisco, Calif; b Department of Vascular Surgery, Kaiser Permanente Northern California, Oakland, Calif; c Department of Critical Care Medicine, Kaiser Permanente Northern California, Oakland, Calif

**Keywords:** Peripheral artery disease, Diabetic acidosis, SGLT-2 inhibitors, Postoperative complication

## Abstract

New pharmacologic advances in the treatment of diabetes include SGLT-2 inhibitors, which have been demonstrated in randomized-controlled clinical trials to reduce overall and cardiac-specific mortality and slow progression of chronic kidney disease. Euglycemic diabetic ketoacidosis is a rare but life-threatening complication associated with the use of SGLT-2 inhibitors. Here we describe a case of severe euglycemic diabetic ketoacidosis after lower extremity bypass in a patient taking an SGLT-2 inhibitor. Awareness of this potential complication is essential as these novel agents are increasingly used in patients with cardiovascular disease.

Diabetes is a common risk factor for peripheral arterial disease, and poor glycemic control is a significant risk factor for progression to amputation.^1^ Recent pharmacologic advances have produced novel oral antihyperglycemics that demonstrate promise in diabetic patients with cardiovascular disease. Specifically, SGLT-2 inhibitors such as empagliflozin (Jardiance; Boehringer Ingelheim Pharmaceutical, Inc, Ridgefield, Conn) and canagliflozin (Invokana; Janssen, Titusville, NJ) have demonstrated in six randomized-controlled clinical trials to have significant benefits in reduction of cardiovascular death, hospitalizations for heart failure, overall risk of death, and risk of progression of chronic kidney disease in diabetic patients.^2^, ^3^, ^4^, ^5^, ^6^, ^7^ As such, the American Diabetes Association and the American Association of Clinical Endocrinology now recommend that diabetic patients with either known cardiovascular disease or high risk for cardiovascular disease should be started on this class of medications, even in the absence of a need for better glucose control.^8^^,^^9^

As the use of SGLT-2 inhibitors has increased, there have been several case reports of a rare but potentially life-threatening complication called euglycemic diabetic ketoacidosis (DKA) associated with these novel agents.^10^ Patients on SGLT-2 inhibitors may present with symptoms of severe DKA and a high anion gap metabolic acidosis with elevated urinary ketones, despite normal or near-normal glucose levels. Hypotension may also occur from associated hypovolemia.^10^ Euglycemic DKA is difficult to diagnose given the absence of hyperglycemia and may be life threatening if untreated. We present here a case of euglycemic DKA after an otherwise uncomplicated lower extremity bypass procedure. The patient provided written consent for this case report. This publication is exempt from institutional board review.

## Case report

Our patient is a 73-year-old man with diabetes, hypertension, coronary artery disease, congestive heart failure, and chronic renal insufficiency (creatinine 1.4 mg/dL) who presented with a nonhealing wound on his left lateral heel and a toe pressure of 4 mm Hg (Society of Vascular Surgery Wound, Ischemia, and Foot Infection score 2-3-0, stage 4).^11^ His diabetes was poorly controlled (A1c 8.8), and he had recently started empagliflozin in addition to metformin and insulin. Empagliflozin and metformin were held on the morning of surgery, and he underwent left femoral endarterectomy with common and external iliac stenting and left femoral to below-knee bypass using the ipsilateral saphenous vein.

His postoperative course initially followed the expected trajectory, but early in the morning of postoperative day 2, he developed acute-onset delirium with severe metabolic acidosis (pH 7.2, PaCO_2_ of 19 mm Hg, bicarbonate of 8.9 mmol/L, and base deficit of 16.7 mmol/L with anion gap 24 mEq/L). Surprisingly, serum lactate was only mildly elevated (3 mmol/L). He was hypotensive requiring volume resuscitation and low-dose pressor support, despite brisk urine output. Workup did not demonstrate evidence of bleeding, infection, or cardiac event to explain his clinical condition. His serum glucose was relatively normal (170-200 mg/dL), but his urinalysis was significant for high urinary glucose and ketones (>500 mg/dL and 80 mg/dL, respectively). Of note, he had not received any insulin on postoperative day 1 given normal glucose levels.

The treating intensivist was familiar with the risk of euglycemic DKA with SGLT-2 inhibitors and started the patient on an insulin drip, resulting in normalization of the severe acidosis. The patient’s blood pressure and mental status also improved with fluids and correction of the DKA. He was transitioned from insulin drip to subcutaneous insulin the following day, and the remainder of his hospital course was unremarkable. He was discharged home on postoperative day 7 without further complication.

## Discussion

Traditionally, DKA is a complication of type I diabetes where deficient insulin production results in both severe hyperglycemia and increased hepatic production of ketone bodies with associated anion-gap metabolic acidosis. Diagnostic criteria generally require glucose over 250 mg/dL, and patients often present with glucose upward of 400 mg/dL.^12^ As such, diagnosis of DKA in a type II diabetic patient with near-normal glucose levels is unexpected.

SGLT-2 inhibitors have several described mechanisms. They function to increase urinary glucose excretion and have both direct and indirect effects to increase glucagon release from pancreatic alpha cells. They also increase hepatic ketogenesis, which shifts metabolism from glucose and lipids as primary fuel sources toward ketone bodies; this has been proposed to be partially responsible for the positive effects on cardiac and renal function. The benefits of SGLT-2 inhibitors have been established through more than six randomized-controlled clinical trials that included almost 47,000 patients with type II diabetes.^2^, ^3^, ^4^, ^5^, ^6^, ^7^ In a recent meta-analysis of the randomized-controlled trials on SGLT-2 inhibitors, there was an overall reduction in the risk of major adverse cardiac events, both for patients with known cardiovascular disease and those without (hazard ratio [HR]: 0.90), cardiovascular-specific death (HR: 0.85), all-cause mortality (HR: 0.87), hospitalizations for heart failure (HR: 0.68), and progression of renal dysfunction (HR: 0.62).^13^ The composite outcome of hospitalizations with heart failure and cardiovascular death was reduced by 22% taking an SGLT-2 inhibitor in patients with and without known cardiac disease. DKA complications were rare in these trials. The CREDENCE study on canagliflozin had the highest incidence, with 2.2 events per 1000 patient-years,^13^ and later analyses reported a lower incidence of 0.522 events per 1000 patient-years.^14^

Most of the data available on euglycemic DKA related to SGLT-2 inhibitors are from case reports; mechanism and risk factors are poorly understood. In one review of 34 cases of euglycemic DKA in association with an SGLT-2 inhibitor, the majority of patients were found to have a precipitating factor before the onset of acidosis: 28% of patients were in the early postoperative period from major surgery, 12% had a major illness, and 28% were in the setting of stopping or reducing their insulin dose.^15^ Despite the classic teaching that DKA is primarily a problem for type I diabetics, 73.5% of patients in this review were type II diabetics.

Treatment of this unusual phenomenon is similar to classic DKA—supportive care with fluid resuscitation and insulin. However, management is challenging as diagnosis is not intuitive and treatment may be delayed or neglected. Initiation of appropriate treatment for euglycemic DKA in a timely manner requires knowledge of its association with SGLT-2 inhibitors and a high index of suspicion. Altered mentation and low CO_2_ on routine metabolic panels should spur further workup with arterial blood gas or urinalysis for patients on SGLT-2 inhibitors. Because vascular surgery patients are nil per os before surgery—often with reduced insulin regimens to avoid hypoglycemia—and undergo long procedures with significant metabolic stress, they may be particularly vulnerable to euglycemic DKA. Our patient’s operation was of expected difficulty and duration, but delayed to late afternoon, the only potential risk factor we identified.

It is essential that vascular surgeons appreciate the possibility of this complication, as use of SGLT-2 inhibitors provides long-term cardiovascular benefits and will become increasingly used. Given the rarity, multicenter data are needed to expand our understanding of preventative factors to support any practice changes. Potential considerations include holding SGLT-2 inhibitors for 48 hours before surgery, which is unproven but suggested based on their average half-life of 12 hours,^15^ and emphasizing early postoperative nutrition with continuation of basal insulin dosing.

## Conclusions

SGLT-2 inhibitors are broadly recommended in diabetic patients with cardiovascular risk factors. Euglycemic DKA is a rare, potentially life-threatening complication associated with the use of SGLT-2 inhibitors. Diagnosis is challenging without hyperglycemia, and awareness of this potential complication is essential to facilitate proper treatment, especially in the postoperative period.
